# A Pediatric Case of Refractory Torsades de Pointes in Autoimmune Hypothyroidism

**DOI:** 10.1210/jcemcr/luae124

**Published:** 2024-07-15

**Authors:** Sri Nikhita Chimatapu, Jessica L Schachter, Anjan S Batra, Rachel Sirignano, Erin R Okawa

**Affiliations:** University of California, Los Angeles, Mattel Children's Hospital, Los Angeles, CA 90095, USA; Department of Cardiology, University of California Irvine Medical Center, Orange, CA 92868, USA; Department of Cardiology, University of California Irvine Medical Center, Orange, CA 92868, USA; Children's Heart Institute, Memorial Care Miller Children's & Women's Hospital, Long Beach, CA 90806, USA; University of California, Los Angeles, Mattel Children's Hospital, Los Angeles, CA 90095, USA

**Keywords:** hypothyroidism, ventricular arrhythmia, Torsades de Pointes, polymorphic ventricular tachycardia, Brugada syndrome

## Abstract

Hypothyroidism can have a significant impact on cardiac contractility, vascular resistance, blood pressure, and cardiac rhythm. Ventricular arrhythmias induced by hypothyroidism are infrequently reported, especially in pediatric cases. A 15-year-old girl with autoimmune hypothyroidism experienced pulseless ventricular arrhythmias on 2 separate occasions because of nonadherence to levothyroxine medication. Subsequent investigations revealed an *SCN5A* mutation associated with Brugada syndrome. A loop recorder captured polymorphic ventricular tachycardia (PMVT), specifically Torsades de Pointes during her second event. Both arrhythmias were addressed only after stabilizing her thyroid hormone levels with replacement therapy. Although rare, patients with uncontrolled hypothyroidism may present with ventricular arrhythmias, particularly PMVT. The cornerstone of treatment for hypothyroidism-induced ventricular arrhythmia is thyroid replacement therapy. The identification of an *SCN5A* mutation unmasked by overt hypothyroidism emphasizes the need for a comprehensive cardiac evaluation in patients with hypothyroidism being assessed for PMVT.

## Introduction

Hypothyroidism is a common endocrine disorder affecting 4% to 10% of the population; it can be detrimental to the cardiovascular system, contributing to cardiac function and rhythm disturbances (1). Electrocardiographic (ECG) changes commonly observed with hypothyroidism include sinus bradycardia, low voltage, atrioventricular block, and prolonged QT (2). When QT prolongation occurs, there is an increased risk of polymorphic ventricular tachycardia (PMVT) secondary to acquired Torsades de Pointes (TdP).

Although Brugada syndrome (BrS) is primarily an inherited condition, a few case studies note a correlation between uncontrolled hypothyroidism and a Brugada pattern on ECG. This may be secondary to the effect of uncontrolled hypothyroidism on myocardial ion channels, which is the pathophysiologic premise for the typical ECG pattern seen in BrS (3‐5). The finding of a classic type I Brugada pattern ECG is associated with increased susceptibility to ventricular arrhythmias and sudden cardiac death (6). We present a unique case of acquired PMVT in a young patient with autoimmune hypothyroidism who was nonadherent to thyroid replacement, and, subsequently, *SCN5A* was discovered.

## Case Presentation

The patient is a 15-year-old female who was initially referred to outpatient pediatric endocrinology at age 12 years with complaints of fatigue, dry skin, facial puffiness, and increased sensitivity to cold before presenting to our facility. She had also demonstrated poor linear growth in the year before her presentation. Initial blood work showed an elevated TSH level of >150 uIU/L [mIU/L] (reference range, 0.50-4.30), a low free T4 (FT4) level of 2.57 pmol/L [0.2 ng/dL] (reference range, 0.9-1.4 ng/dL), and a positive thyroglobulin antibody level of >1000 IU/mL (reference range, <1 IU/mL). Her thyroid peroxidase antibody and thyroid-stimulating immunoglobulin were negative. Her vitals were within normal limits, but her thyroid examination revealed mild diffuse enlargement. Hence, she was diagnosed with autoimmune hypothyroidism and started on 75 mcg of levothyroxine daily.

After her diagnosis, the patient saw her pediatric endocrinologist sporadically and was often noted to be skipping several doses of levothyroxine. Her nonadherence was also reflected biochemically, with her TSH being persistently elevated and FT4 being below normal. As a result, her levothyroxine dose was serially increased to 125 mcg by her pediatric endocrinologist. Her last blood work 6 months before presenting to our facility showed an elevated TSH of 105.46 IU/L [mIU/L] and a low FT4 of 10.2 pmol/L [0.8 ng/dL]. Furthermore, during these 3 years, the patient gained 57 lb and was diagnosed with prediabetes as her hemoglobin A1c became elevated to 5.7% (reference range, <5.6%). She was started on metformin by her pediatric endocrinologist but was not adherent to this medication either.

## Diagnostic Assessment

On the day of her initial presentation to our facility, the patient experienced a syncopal episode during her physical education class. She reportedly did not have a pulse on the field. Hence, cardiopulmonary resuscitation was started, and an automated external defibrillator was placed. The automated external defibrillator showed a shockable rhythm, and the patient was cardioverted on the field, following which she regained consciousness. She was then brought to our emergency department where she was noted to have severe hypothyroidism. Her TSH was 387.400 uIU/L [mIU/L] (reference range, 0.463-3.980 uIU/mL), FT4 was 1.67 pmol/L [0.13 ng/dL] (reference range, 0.78-1.33 ng/dL), and free T3 was <0.76 pmol/L [<0.50 pg/mL] (reference range, 2.91-4.53 pg/mL). Her vitals on arrival were stable but the physical examination revealed a sallow appearance. The rest of the physical examination was reported to be normal including the thyroid examination. Her initial ECG demonstrated a prolonged QTc of 494 ms.

## Treatment

She was then started on 100 mg of hydrocortisone every 6 hours; this was discontinued after 24 hours due to a robust baseline cortisol level of 744.83 nmol/L [27 mcg/dL]. At the same time, she was started on 100 mcg of levothyroxine IV, which was increased to 125 mcg the next day. She was also started on 25 mcg twice daily of liothyronine. On day 3 of this admission, levothyroxine IV was switched to 150 mcg orally and her thyroid function tests improved with TSH 149.2 uIU/mL [mIU/L], FT4 4.37 pmol/L [0.34 ng/dL], and free T3 1.06 pmol/L [0.69 pg/mL]. Her ECG showed improvement in QTc to 395 ms with treatment of her hypothyroidism. Her cardiac magnetic resonance imaging showed an ejection fraction of 40% (normal, 55%-65%) with no evidence of myocardial fibrosis, scarring, or arrhythmogenic right ventricular dysplasia. Implantable cardioverter defibrillator placement was deferred for an implantable loop recorder until further outpatient evaluation was completed. Genetic testing for cardiac channelopathies was sent and returned positive for BrS with a pathogenic *SCN5A* mutation. At the time of discharge, her TSH was 30.8 uIU/mL [mIU/L], FT4 was 6.95 pmol/L [0.54 ng/dL], and total T3 was 4.15 nmol/L [270 ng/dL] (reference range, 80-213 ng/dL). Given that her total T3 was elevated, liothyronine was discontinued and she was discharged home on 150 mcg of levothyroxine to follow up with her outpatient pediatric endocrinologist. Notably, her baseline ECG postthyroid replacement did not demonstrate a Brugada pattern.

**Figure 1.**
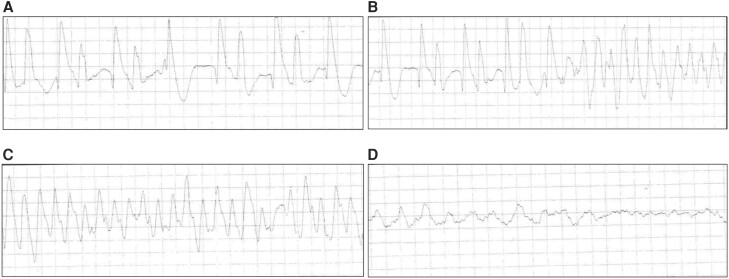
Loop recorder tracings at the time of syncope and cardiac arrest. (A) Inciting rhythm shows ventricular bigeminy. (B) Demonstration of bigeminy propagating ventricular tachycardia. (C) Polymorphic ventricular tachycardia, in this case, Torsades de Pointes. (D) Ventricular tachycardia degenerating into ventricular fibrillation.

The patient was unable to reestablish care with her pediatric endocrinologist after this initial hospitalization and presented again 8 months later after a cardiac arrest at home. She was subsequently found to have severe hypothyroidism with an elevated TSH of 403 uIU/mL [mIU/L] and an undetectable FT4. On arrival at our emergency department, she remained in full cardiac arrest and was noted to have pulseless monomorphic ventricular tachycardia on ECG. Cardiopulmonary resuscitation was continued with further defibrillation and administration of PALS medications with eventual return of spontaneous circulation after 48 minutes. She was then intubated and started on 100 mcg of levothyroxine IV daily along with continuous levothyroxine at 5 mcg/kg/h (120 mcg daily). Despite having a previously robust cortisol, she was given 40 mg of hydrocortisone every 6 hours because of her precarious clinical status. Her ECG showed a wide complex tachycardia at 112 beats per minute with prolonged QTc >500 ms and after return of spontaneous circulation showed a wide complex ventricular escape rhythm at 50 beats per minute and a QTc of 448 ms. There was no evidence of a Brugada pattern. Review of her loop recorder revealed initial ventricular bigeminy becoming TdP followed by ventricular fibrillation (VF) (Fig. 1). She had a bedside transvenous pacemaker placed but attempts to overdrive pace could not completely suppress her ventricular arrhythmias. Her echocardiogram showed moderately decreased left ventricular function with severe hypokinesia of the apex. Although not indicated at that time, given the concern for an impending requirement of extracorporeal membrane oxygenation, she was transferred to a higher level of care.

By the time of transfer, the patient had stabilized sufficiently to discontinue the hydrocortisone and decrease the levothyroxine. The patient received 75 mcg of levothyroxine IV daily and transitioned to 112.5 mcg levothyroxine orally as her thyroid function tests showed improvement with TSH 205 uIU/mL[mIU/L] and FT4 9.2 pmol/L[0.72 ng/dL]. She did not receive extracorporeal membrane oxygenation.

On return to our facility, TSH was 144.2 uIU/mL [mIU/L] and FT4 was 10.1 pmol/L [0.79 ng/dL]; therefore, the oral levothyroxine dose was increased to 125 mcg. During this hospitalization, her TSH was noted to be up-trending to 156.5 uIU/mL [mIU/L]. Given the concern for poor absorption of levothyroxine, liothyronine was added once again to her regimen (5 mcg daily). This was later discontinued. The patient then returned to her baseline health including baseline neurologic function and underwent an electrophysiologic study with procainamide challenge to evaluate for phenotypic BrS, which was negative. Following this, she had a transvenous automatic implantable cardioverter-defibrillator placed for secondary prevention. Her TSH before discharge was 2.895 uIU/mL [mIU/L]; she had a mildly elevated FT4 of 20.3 pmol/L [1.58 ng/dL] but continued the same levothyroxine dose of 125 mcg.

## Outcome and Follow-up

Because her presentations were due to medication nonadherence, we implemented educational programs, reminders, and multidisciplinary support and collaborated with her family to ensure a supportive environment to prevent further admissions. She is currently on 112 mcg as her most recent blood work during a follow-up outpatient visit revealed a TSH of 0.16 uIU/mL [mIU/L], FT4 of 18.02 pmol/L [1.4 ng/dL], and total T3 of 2.76 nmol/L [176 ng/dL].

## Discussion

Hypothyroidism is a well-known cause of various cardiovascular abnormalities, including ventricular tachycardia. Possible proposed mechanisms for ventricular arrhythmia include T3's interaction with electrophysiological properties of the myocardium. Low T4 levels in hypothyroidism, subsequently lead to low T3, which then causes prolongation of the QT interval and dispersion of ventricular repolarization (7). Although ECG changes are common in this disease process, the incidence rates of ventricular arrhythmias and ventricular tachycardia in hypothyroidism are 6.58% and 2.63%, respectively (8). Kandan et al presented an 85-year-old female with lightheadedness secondary to TdP with prolonged QT interval. Despite stopping offending agents, including digoxin and hydroxychloroquine, she continued to have recurrent arrhythmias and prolonged QT and was subsequently diagnosed with hypothyroidism. Treatment with levothyroxine corrected her arrhythmia and ECG abnormalities (9‐11). Similarly, Shojaie et al described a case of severe hypothyroidism manifesting with syncope and PMVT that responded well only after treatment with levothyroxine (10).

For the management of hypothyroidism-induced ventricular tachycardia, immediate correction of thyroid levels is essential, typically starting with IV levothyroxine, followed by maintenance on oral levothyroxine (12). It is crucial to initiate thyroid replacement therapy cautiously in patients with severe hypothyroidism and preexisting heart conditions to avoid exacerbating heart failure or angina. Clinicians should also be aware of the potential delay between thyroid hormone administration and its genomic effects, adjusting treatment expectations accordingly. While awaiting normalization of FT4 levels, temporizing measures with cardioversion/defibrillation, antiarrhythmics, and overdrive pacing are typically attempted. When choosing antiarrhythmic therapy, caution must be used to not further exacerbate arrhythmias. Amiodarone in particular can further prolong the QT interval, which may potentiate TdP whereas lidocaine can shorten the QT interval (13). In our case, the patient continued to have pulseless episodes of TdP despite being on an IV levothyroxine drip with push doses. Antiarrhythmic therapy and pacing were also unable to control her ventricular arrhythmia until the normalization of FT4 levels.

BrS is a life-threatening genetic disorder with 20% to 30% cases involving *SCN5A* mutations that affect the Nav1.5 channel crucial for cardiac action potential propagation (14). These mutations can lead to different types of sodium currents, displaying characteristics of multiple cardiac syndromes, known as overlap syndromes (15). Typically, *SCN5A* variants show autosomal dominant traits that may cause dominant-negative effects or haploinsufficiency, altering ion conductance (14, 16). The genetic landscape of BrS also shows heterogeneity with other genes contributing to its phenotype (17). Nongenetic factors like hypothyroidism can induce arrhythmias in patients with BrS (6) because thyroid dysfunctions are linked to ECG changes resembling Brugada patterns, reversible with thyroid level normalization (3‐5). Although these patients typically do not show ventricular tachycardia or TdP, hypothyroidism exacerbates the arrhythmogenic risk in BrS, especially in those with *SCN5A* mutations. These mutations, coupled with thyroid conditions such as thyrotoxicosis (18) or hypothyroidism (19), increase the likelihood of ventricular arrhythmias in this high-risk group. Thus, thyroid states can crucially influence *SCN5A* function, heightening the arrhythmogenic potential in BrS patients.

Ventricular tachycardia is an uncommon but significant complication of severe hypothyroidism, emphasizing the need for endocrinologists to recognize this critical association. Prompt treatment of the underlying hypothyroid condition is essential for managing these arrhythmias. Furthermore, endocrinologists should routinely evaluate thyroid function in patients with suspected or established BrS or presenting with long QT/PMVT, given the potential impact of hypothyroidism on *SCN5A* gene expression and its arrhythmogenic consequences.

## Learning Points

- Ventricular tachycardia is an uncommon yet severe condition that may occur in cases of uncontrolled hypothyroidism.
- Prompt treatment of the underlying hypothyroidism is the key to alleviate arrhythmias.
- Health care practitioners should regularly screen for thyroid issues in patients with BrS or prolonged QT/PMVT, as imbalances in thyroid hormones can impact *SCN5A* gene function, increasing the risk of arrhythmias.

## Data Availability

Original data generated and analyzed for this case report are included in this published article.
